# Conditional and constitutive expression of a Tbx1-GFP fusion protein in mice

**DOI:** 10.1186/1471-213X-13-33

**Published:** 2013-08-23

**Authors:** Laina Freyer, Sonja Nowotschin, Melinda K Pirity, Antonio Baldini, Bernice E Morrow

**Affiliations:** 1 Department of Genetics, Albert Einstein College of Medicine, 1301 Morris Park Avenue, Bronx, NY 10461, USA; 2 Departments of Ob/Gyn and Pediatrics, Albert Einstein College of Medicine, 1301 Morris Park Avenue, Bronx, NY 10461, USA; 3 Present address: Department of Developmental Biology, Memorial Sloan-Kettering Cancer Center, 1275 York Avenue, New York, NY 10065, USA; 4 Present address: Institute of Genetics, Biological Research Centre, Hungarian Academy of Sciences, Temesvari krt 62, H-6726, Szeged, Hungary; 5 Institute of Genetics and Biophysics, Via Pietro Castellino 111, 80131, Napoli, Italy

**Keywords:** VCFS/DGS, Tbx1, Rosa26, Mouse model, Gain-of-function

## Abstract

**Background:**

Velo-cardio-facial syndrome/DiGeorge syndrome (VCFS/DGS) is caused by a 1.5-3 Mb microdeletion of chromosome 22q11.2, frequently referred to as 22q11.2 deletion syndrome (22q11DS). This region includes *TBX1*, a T-box transcription factor gene that contributes to the etiology of 22q11DS. The requirement for *TBX1* in mammalian development is dosage-sensitive, such that loss-of-function (LOF) and gain-of-function (GOF) of *TBX1* in both mice and humans results in disease relevant congenital malformations.

**Results:**

To further gain insight into the role of Tbx1 in development, we have targeted the *Rosa26* locus to generate a new GOF mouse model in which a Tbx1-GFP fusion protein is expressed conditionally using the Cre/LoxP system. *Tbx1-GFP* expression is driven by the endogenous *Rosa26* promoter resulting in ectopic and persistent expression. *Tbx1* is pivotal for proper ear and heart development; ectopic activation of *Tbx1-GFP* in the otic vesicle by *Pax2-Cre* and *Foxg1-Cre* represses neurogenesis and produces morphological defects of the inner ear. Overexpression of a single copy of *Tbx1-GFP* using *Tbx1*^*Cre/+*^ was viable, while overexpression of both copies resulted in neonatal lethality with cardiac outflow tract defects. We have partially rescued inner ear and heart anomalies in *Tbx1*^*Cre/-*^ null embryos by expression of *Tbx1-GFP*.

**Conclusions:**

We have generated a new mouse model to conditionally overexpress a GFP-tagged Tbx1 protein *in vivo*. This provides a useful tool to investigate *in vivo* direct downstream targets and protein binding partners of Tbx1.

## Background

Velo-cardio-facial syndrome/DiGeorge syndrome (VCFS/DGS), also known as 22q11.2 deletion syndrome (22q11DS), is the most common microdeletion syndrome occurring *de novo* in approximately 1/4,000 live births [1]. *TBX1*, encoding a T-box transcription factor, is located within the 1.5 Mb critically deleted region and haploinsufficiency of this gene is responsible for the congenital defects associated with 22q11DS [2]. *Tbx1* null mutant mice have malformations of the heart, thymus/parathyroid, craniofacial region, and ear that are similar but more severe, to what is typically found in 22q11DS patients [2-4]. Previously generated BAC316.23 transgenic mice expressing 8–10 copies of human *TBX1* also have developmental defects of the same tissues affected in *Tbx1*^*−/−*^ null mice, resembling clinical features of a recently identified 22q11 duplication syndrome [5-9]. Ablation studies using *Tbx1*^*flox*^ conditional null mutants have proven to be indispensible for understanding the tissue and cell-type specific roles of the *Tbx1* gene [10,11]. COET, a transgenic mouse line with conditional overexpression of Tbx1, has previously been reported [12]. However, the COET mouse line does not contain a protein affinity tag on the Tbx1 protein. We have generated a conditional allele targeted at the *Rosa26* locus for the purpose of expressing additional copies of *Tbx1* fused to a GFP reporter in a tissue-specific manner. We chose a GFP tag in part to detect live GFP as a readout for activation of Tbx1-GFP *in vivo* and to use it for future biochemical or chromatin immunoprecipitation experiments. *Tbx1* mutant rescue experiments demonstrate that Tbx1-GFP fusion protein functions *in vivo* in a manner similar to that of endogenous Tbx1. The *Tbx1-GFP* mouse allele provides a new gain-of-function model that is targeted to the constitutively active *Rosa26* locus; targeting *Tbx1-GFP* to the endogenous *Tbx1* locus would simultaneously disrupt the normal function of the targeted allele (as is the case for *Tbx1*^*Cre/+*^ mice, Huynh et al., 2007). As such, our model works to activate 1–2 ectopic copies of Tbx1 while simultaneously tracing cells with GFP, and should therefore be of high value in future experimental studies.

## Results and discussion

**Figure 1:**
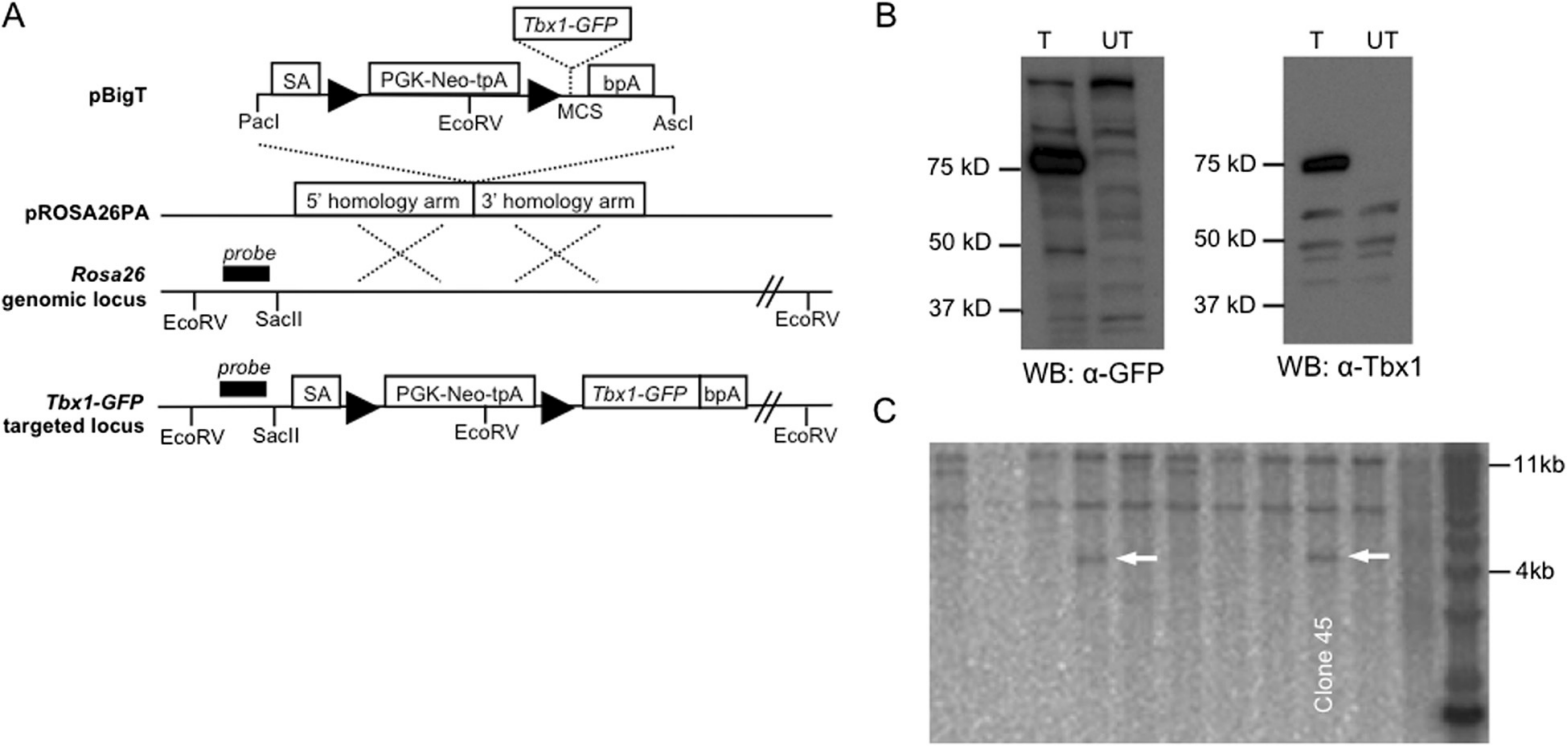
**Generation of the *Tbx1-GFP *mouse. (A)** Tbx1-GFP was inserted into the multiple cloning site (MCS) of pBigT downstream of a triple polyadenylation sequence (tpA) that is flanked by LoxP sites (triangles). Additional subcloning into the pROSA26PA plasmid resulted in the final targeting construct. 5’ and 3’ homology arms mediate recombination with the endogenous *Rosa26* locus. **(B)** Whole cell lysates from COS7 cells transfected (T) with Tbx1-GFP plasmid or untransfected (UT). The fusion protein is ≈75 kD and is detected by anti-GFP and anti-Tbx1 on Western blot. **(C)** Southern blot was used to screen ESC clones. Genomic DNA was linearized with EcoRV and hybridized with a radiolabeled probe that binds upstream of the 5’ homology arm. Two of five positive clones are shown. The correctly targeted allele is 4,071 bp and the wildtype allele is 11,517 kb.

To generate the *Tbx1-GFP* allele, we first generated a bacterial plasmid that expresses a Tbx1-GFP fusion protein of approximately 75 kilodaltons (kD) (Figure 1A), corresponding to the combined mass of Tbx1 (~50 kD) and GFP (~27 kD) (Figure 1B). The *Tbx1-GFP* fragment was inserted downstream of a triple polyadenylation (tpA) transcriptional stop sequence that is flanked by LoxP sites in the pBigT vector [13]. The loxP-tpA-loxP-Tbx1-GFP element was cloned into the pROSA26PA vector for targeting to the *Rosa26* locus [13,14], and electroporated into WW6 (129/SvJ) mouse embryonic stem cells (ESC). We identified 5/56 correctly targeted ESC clones by Southern blot analysis (Figure 1C). Clone 45 was chosen for blastocyst injection in C57BL6 females to generate chimeras for germline transmission.

**Figure 2:**
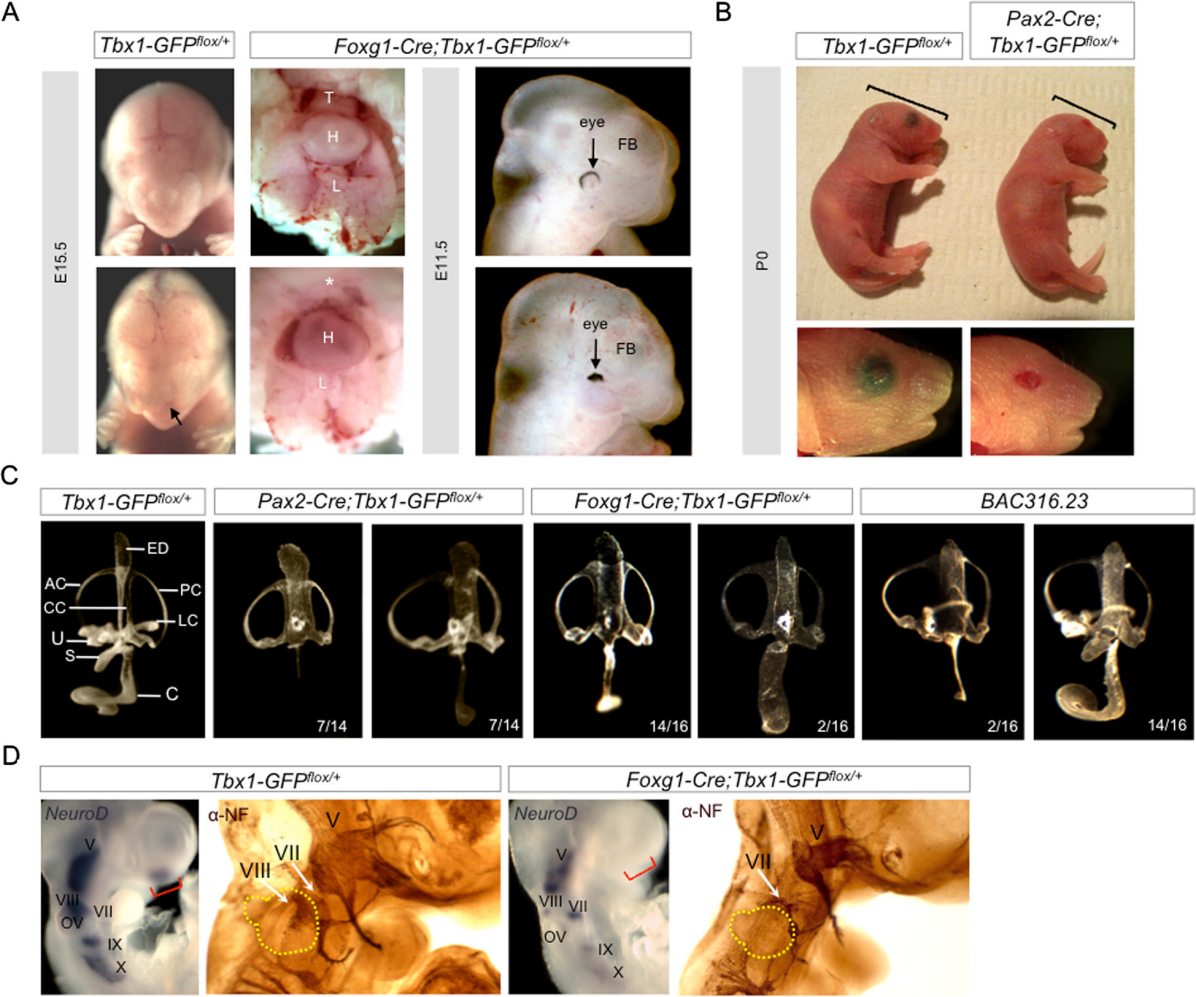
**Activation of Tbx1-GFP by *Foxg1-Cre *and *Pax2-Cre*. (A)***Foxg1-Cre;Tbx1-GFP*^*flox/+*^ mutants have thymic aplasia and nasal malformations at E15.5, and eye and pharyngeal defects at E11.5. **(B)***Pax2-Cre;Tbx1-GFP*^*flox/+*^ mutants die shortly after birth. They have microcephaly and ocular defects. Thymus (T), heart (H), lungs (L), forebrain (FB). **(C)** Paintfilling of inner ears in *Pax2-Cre;Tbx1-GFP*^*flox/+*^ and *Foxg1-Cre;Tbx1-GFP*^*flox/+*^ mutants. The endolymphatic duct (ED) and common crus (CC) are enlarged. The lateral semicircular canal (LC), utricle (U), and saccule (S) are missing. The cochlea (C) is shortened to varying degrees. BAC316.23 mice show similarities in inner ear morphological defects. Anterior canal (AC), posterior canal (PC). **(D)** Wholemount RNA *in situ* hybridization at E9.5 for *NeuroD*, expressed in neuroblasts of the cranial ganglia. Wholemount immunohistochemistry stains cranial sensory ganglia with anti-Neurofilament (NF) at E10.5. Red brackets indicate olfactory placode. Otic vesicle is circled in yellow.

To test the *Tbx1-GFP* allele, we crossed *Tbx1-GFP*^*flox/flox*^ mice to *Foxg1-Cre* and *Pax2-Cre* mice [15,16] and analyzed their respective phenotypes. Both Cre drivers have previously been used to inactivate Tbx1 by tissue-specific recombination of loxP sites [11,17]. *Foxg1-Cre* expression overlaps with endogenous *Tbx1* expression in tissues such as the pharyngeal apparatus and otic vesicle (OV) [11,15,17]. In the study presented here, we show that *Foxg1-Cre* can ectopically activate *Tbx1-GFP* in the olfactory placode, forebrain, and optic vesicle resulting in morphological defects of these tissues during development (Figure 2A). In addition, by E15.5, *Foxg1-Cre;Tbx1-GFP*^*flox/+*^ mutants display thymic aplasia likely due to overexpression of *Tbx1-GFP* in the 3^rd^ pharyngeal pouch, and abnormal formation of the nasal prominence. *Pax2-Cre;Tbx1-GFP*^*flox/+*^ mutants are perinatal lethal (Figure 2A).

Since inner ear and cardiac defects are two of the most prominent features in the *Tbx1* null mutant, we decided to examine those structures in more detail in the *Pax2-Cre;Tbx1-GFP*^*flox/+*^ mutants and *Foxg1-Cre;Tbx1-GFP*^*flox/+*^ mutants, respectively. *Foxg1-Cre* and *Pax2-Cre* are both strongly expressed throughout the OV, from which the inner ear forms. Both loss or gain of *Tbx1* function in the OV disrupts inner ear morphogenesis [5,18,19]. Paintfilling of the inner ear in *Foxg1-Cre;Tbx1-GFP*^*flox/+*^ and *Pax2-Cre;Tbx1-GFP*^*flox/+*^ mutants at E14.5 shows an enlarged endolymphatic duct (ED) and common crus (CC) that joins the anterior and posterior semicircular canals (SCC) (Figure 2B). The utricle, saccule, and lateral SCC are missing. The cochlea is hypoplastic with varying degree, but enlarged in two instances (Figure 2B). This phenotype is very similar to that of BAC316.23 transgenic mice [5]; with the exception that BAC316.23 mice still possess the lateral SCC, and in most cases the saccule and utricle are present, albeit malformed (Figure 2B).

*Tbx1* is also known to repress neurogenesis of the VIIIth cranial ganglion that innervates the inner ear [19]. Here we show that in *Foxg1-Cre;Tbx1-GFP*^*flox/+*^ mutants, expression of *Neurogenic differentiation factor 1* (*NeuroD*) is nearly abolished in the VIIIth cranial ganglion, and highly attenuated in cranial ganglia V, VII, IX, and X (Figure 2C). Immunohistochemistry with anti-Neurofilament confirmed loss of the VIIIth cranial ganglion and abnormal size and misguided projections from other cranial ganglia in *Foxg1-Cre;Tbx1-GFP*^*flox/+*^ mutants (Figure 2C). In addition, we found that *NeuroD* expression is completely missing from the olfactory placode of *Foxg1-Cre;Tbx1-GFP*^*flox/+*^ mutants; a region where *Foxg1-Cre* is active and there is no *Tbx1* expression in a wild type context suggesting that gain of function of Tbx1 in the olfactory placode leads to ectopic repression of *NeuroD*. These findings demonstrate that Tbx1 could act directly or indirectly on neurogenic factors that are common to both otic and olfactory placodes.

**Figure 3:**
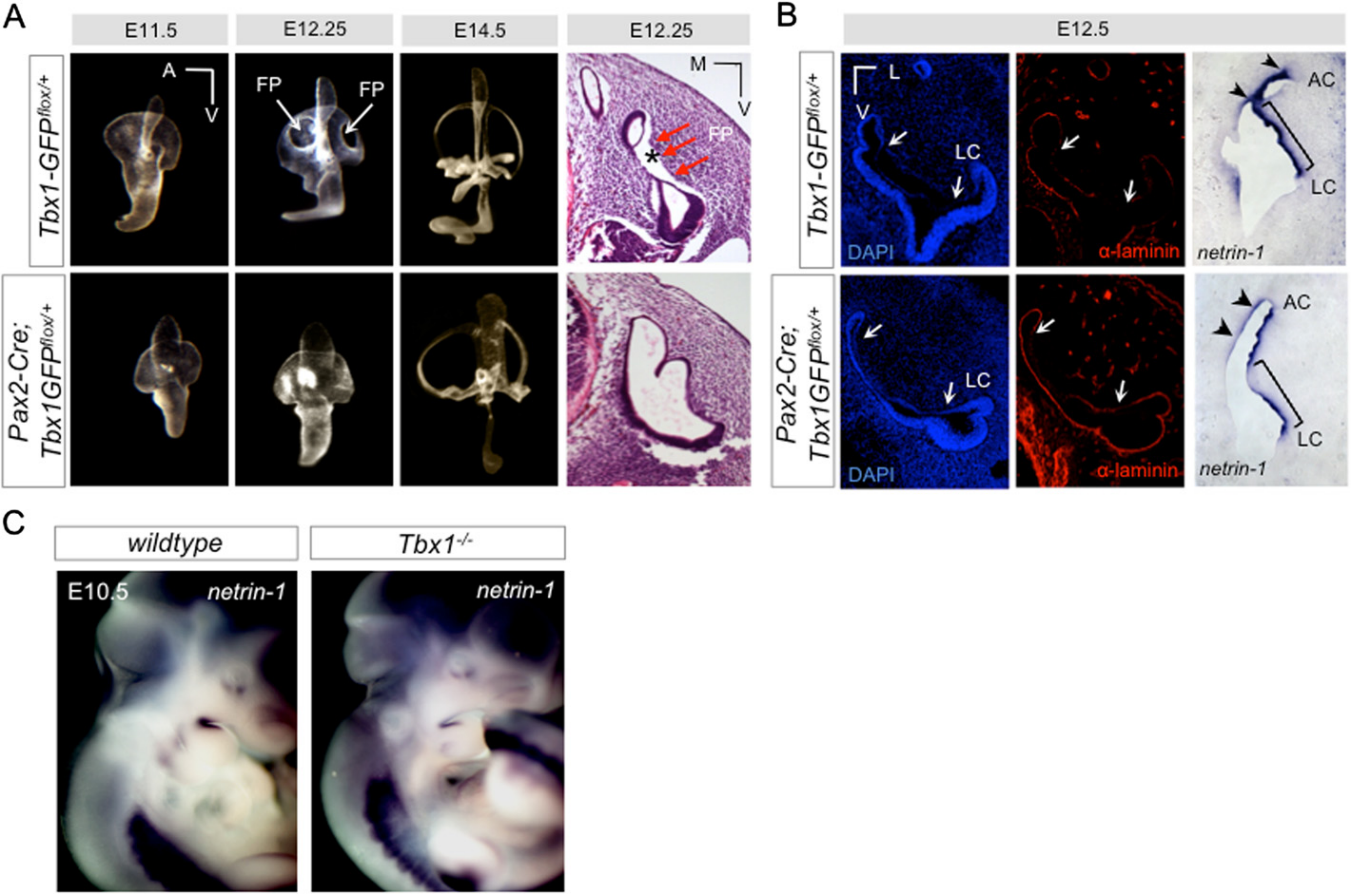
**Altered cell adhesion properties in *Tbx1 *mutants. (A)** Paintfilling of inner ears from *Pax2-Cre;Tbx1-GFP*^*flox/+*^ mutants at progressively earlier stages of development reveals a delay in SCC formation. At E12.25, fusion plates (arrows, FP) are visible in the controls, but do not appear to have formed in the mutants. Histological analysis confirms defects in the fusion plates (FP, red arrows). In controls, the vestibular epithelia can be seen to have separated from the mesenchyme (asterisks). In *Tbx1-GFP* mutants, there is no separation of the epithelium from surrounding tissue. **(B)** Immunofluorescence with anti-laminin (red). Arrows indicate the span of the fusion plate epithelia. There is more intact laminin in the *Tbx1-GFP* mutants. RNA *in situ* hybridization to *netrin-1* on sections. *Netrin-1* expression is reduced in the fusion plates of *Tbx1-GFP* mutants. Anterior canal (AC, lateral canal (LC). **(C)** Expression of *netrin-1* mRNA on wildtype and *Tbx1*^*−/−*^ null mice. *Netrin-1* expression is increased in the mesenchyme surrounding the inner ear in the absence of endogenous *Tbx1*.

Examination of SCC defects in the *Pax2-Cre;Tbx1-GFP*^*flox/+*^ mutants using paintfilling revealed a delay in SCC formation at earlier developmental stages (Figure 3A). In the wild type, clearing of the fusion plates is visible by E12.25 and complete by E12.5 [20]. In *Pax2-Cre;Tbx1-GFP*^*flox/+*^ mutants, the fusion plates have not yet joined at E12.25 although they appear to partially recover by E14.5 (Figure 3A). Histological sections at E12.25 confirm that the fusion plates in *Pax2-Cre;Tbx1-GFP*^*flox/+*^ mutants remain attached to the periotic mesenchyme compared to control littermates (Figure 3A). We examined expression of laminin and *netrin-1*, a secreted protein that is related to laminins and promotes basal lamina breakdown [21,22]. We observed decreased laminin protein levels along the otic epithelium, especially along the site of lateral SCC formation along with a corresponding decrease in *netrin-1* mRNA expression along the fusion plates in *Pax2-Cre;Tbx1-GFP*^*flox/+*^ mutants (Figure 3B). It is possible that *Tbx1* may act to repress *netrin-1*, thereby modulating cell adhesion properties. Complementation of these results occurred in *Tbx1*^*−/−*^ null mice in which expression of *netrin-1* is expanded in the mesenchyme surrounding the OV (Figure 3C). We did not observe expanded *netrin-1* expression in the OV of *Tbx1*^*−/−*^ null mice, possibly due to the severity of the OV phenotype and subsequent absence of vestibular structures.

**Figure 4:**
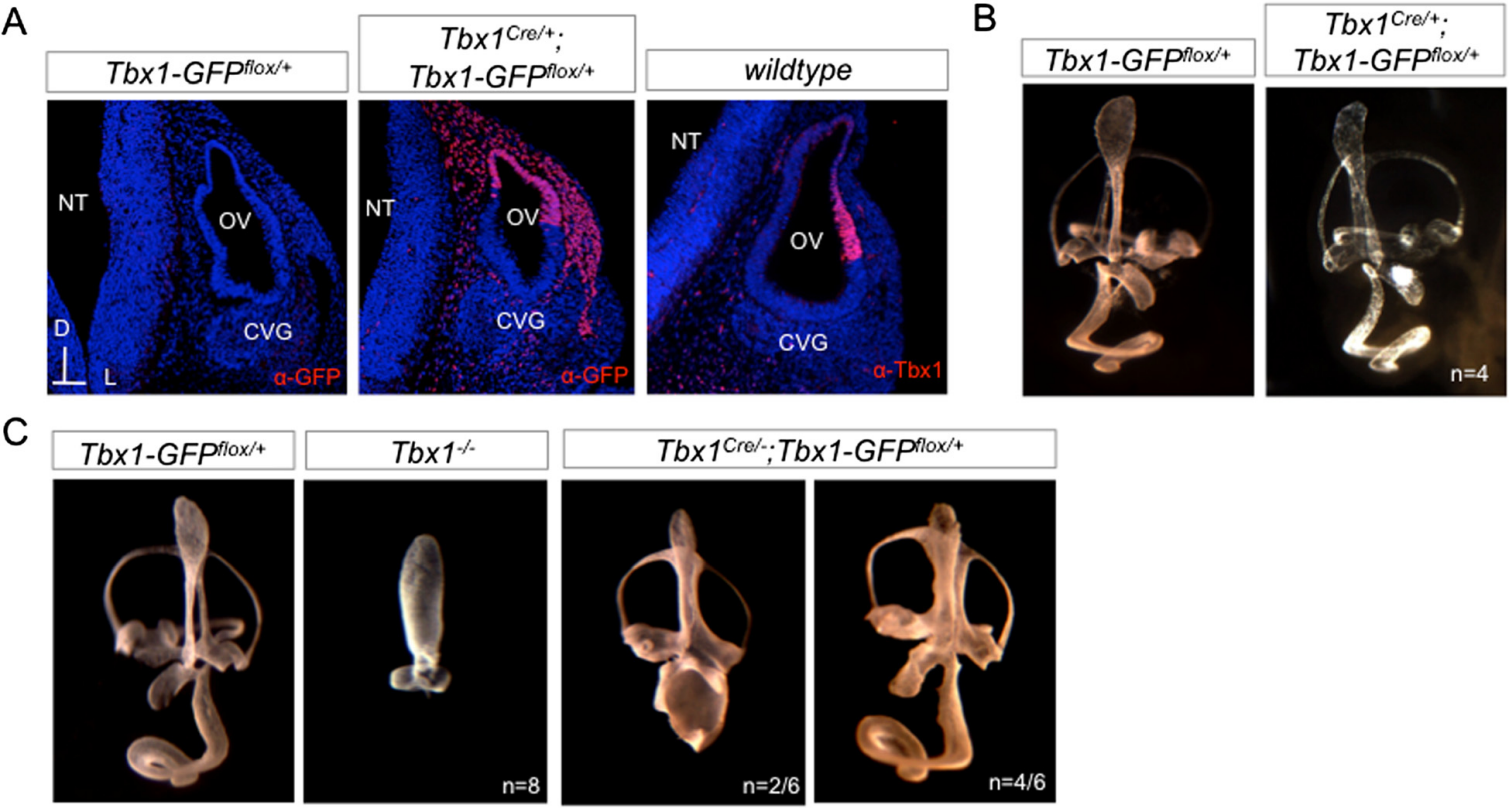
**Activation of *Tbx1-GFP *in the inner ear using *Tbx1***^***Cre/+***^***. *(A)** Immunofluorescence with GFP (red) and Tbx1 (red) antibodies on E10.5 transverse sections through the otic vesicle (OV). The VIIIth cranial ganglion, also known as the cochleovestibular ganglion (CVG) is adjacent to the OV. Neural tube (NT). **(B)** Activation of *Tbx1-GFP* by *Tbx1*^*Cre/+*^ does not produce major morphological defects of the inner ear, as visualized by paintfilling at E14.5. **(C)** Paintfilling shows failure of inner ear morphogenesis in *Tbx1*^*Cre/-*^ null mice at E14.5 compared to the *Tbx1-GFP*^*flox/+*^ control. Activation of a single allele of *Tbx1-GFP* on a *Tbx1*^*Cre/-*^ null background is capable of partially rescuing morphogenesis.

To test whether Tbx1-GFP could rescue inner ear defects that occur in *Tbx1*^*Cre/-*^ null embryos, we crossed *Tbx1-GFP*^*flox/flox*^ mice to *Tbx1*^*Cre/+*^ mice [23]. *Tbx1*^*Cre/-*^ mice have been shown to recapitulate the *Tbx1*^*−/−*^ null phenotype [23]. When Cre is expressed, cells from the *Tbx1*^*Cre/+*^ expressing domain are positive for GFP in a manner that represents the *Tbx1* lineage in the OV in embryos (Figure 4A). Expression of Tbx1-GFP under the *Rosa26* promoter alone is not sufficient for direct visualization of natural GFP, therefore Tbx1-GFP protein was detected using an antibody to GFP. Since expression of *Tbx1-GFP* is persistently driven by the endogenous *Rosa26* promoter, we expect the domain of activated *Tbx1-GFP* to be more extensive than native *Tbx1* expression (Figure 4A). Immunofluorescence with anti-GFP antiserum demonstrated that there is no Tbx1-GFP protein in the absence of Cre expression in the OV (Figure 4A). *Tbx1*^*Cre/+*^*;Tbx1-GFP*^*flox/+*^ mutants did not exhibit gross morphological defects of the inner ear based on paintfilling (Figure 4B), perhaps because one allele of *Tbx1* is removed by knock-in of *Cre*. Paintfilling confirmed that the inner ears of *Tbx1*^*−/−*^ null mice lack all discernible vestibular and auditory structures with the exception of what appears to be an enlarged ED (Figure 4C). When a single allele of *Tbx1-GFP* is activated in a *Tbx1*^*Cre/-*^ background, there is rescue of the anterior and posterior SCCs in all embryos, and partial rescue of the saccule and cochlea (n=6) (Figure 4C). Complete rescue of the null phenotype is not expected with constitutive activation of *Tbx1-GFP* because temporal regulation of *Tbx1* is required for normal inner ear development.

**Figure 5:**
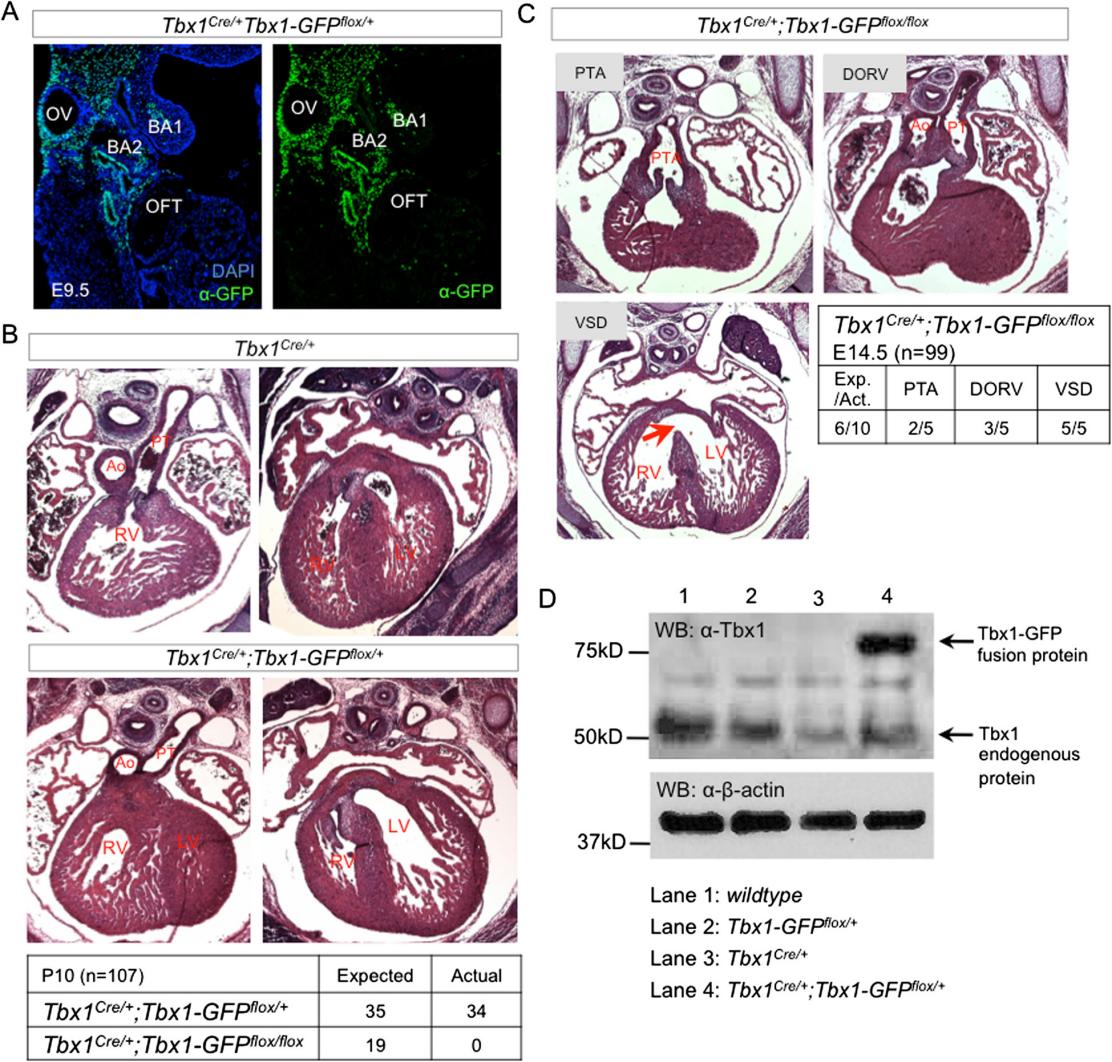
**Dosage effects of *Tbx1-GFP *activation on heart development. (A)** Immunofluorescence with a GFP antibody shows activation of *Tbx1-GFP* by *Tbx1*^*Cre/+*^ at E9.5. GFP is detected in the posterior otic vesicle (OV), 1^st^ and 2^nd^ branchial arches (BA1, BA2), and mesoderm including the secondary heart field proximal to the outflow tract (OFT). **(B)** Histological transverse sections at E14.5 shows that *Tbx1*^*Cre/+*^*;Tbx1-GFP*^*flox/+*^ mutants do not have outflow tract or ventricular defects. They survive in normal mendelian ratios, unlike *Tbx1*^*Cre/+*^*;Tbx1-GFP*^*flox/flox*^ mutants which are not viable. **(C)** Activation of both alleles of *Tbx1-GFP* by *Tbx1*^*Cre/+*^ causes outflow tract septation (persistant truncus arteriosis, PTA) and alignment (double outlet right ventricle , DORV) defects with ventricular septal defect (VSD). **(D)** Western blot of proteins isolated from E9.5 pharyngeal regions to compare protein expression levels of Tbx1-GFP and endogenous Tbx1. For each genotype, we pooled 4 embryos (23–24 somite stage) and loaded equal amounts of protein per lane. The same membrane was probed with antibodies to Tbx1 and β-actin. The expected size of endogenous Tbx1 is ≈50 kD while the Tbx1-GFP fusion protein is ≈75kD. β-actin is detected at ≈40 kD.

The *Tbx1*^*Cre/+*^ allele was also used to activate *Tbx1-GFP* in other endogenous sites of Tbx1 expression such as the pharyngeal mesoderm including the secondary heart field (Figure 5A). Histological analysis of *Tbx1*^*Cre/+*^*;Tbx1GFP*^*flox/+*^ embryos at E14.5 showed normal heart development (Figure 5B). Genotyping of mice at P10 confirmed presence of live *Tbx1*^*Cre/+*^*;Tbx1GFP*^*flox/+*^ mice in normal Mendelian ratios (Figure 5B). In contrast, mice that have both *Tbx1-GFP* alleles activated by *Tbx1*^*Cre*/+^ are not viable (Figure 5B), prompting us to assess the heart phenotype in *Tbx1*^*Cre/+*^*;Tbx1GFP*^*flox/flox*^ embryos at E14.5. *Tbx1*^*Cre/+*^*;Tbx1GFP*^*flox/flox*^ embryos exhibited defects such as persistent truncus arteriosus (PTA, 2/5 embryos) and double outlet right ventricle (DORV, 3/5 embryos), all with ventricular septal defects (VSD, 5/5 embryos) (Figure 5C). Overexpression of *TBX1* has been previously associated with VSD in ~10% of BAC316.23 transgenic mice [2]. Heart malformations can account for the perinatal lethality of *Tbx1*^*Cre/+*^*;Tbx1GFP*^*flox/flox*^ mice. We wanted to compare the protein expression levels of the Tbx1-GFP fusion protein activated in the Tbx1 domain compared to endogenous Tbx1 protein. To do this, we performed western blot on tissue samples (Figure 5D) and observed that the level of Tbx1-GFP protein expressed from a single allele was higher than that observed for endogenous wildtype Tbx1. However, this may reflect the larger domain of expression of Tbx1-GFP within the cell lineage that is labeled by *Tbx1*^*Cre/+*^.

**Figure 6:**
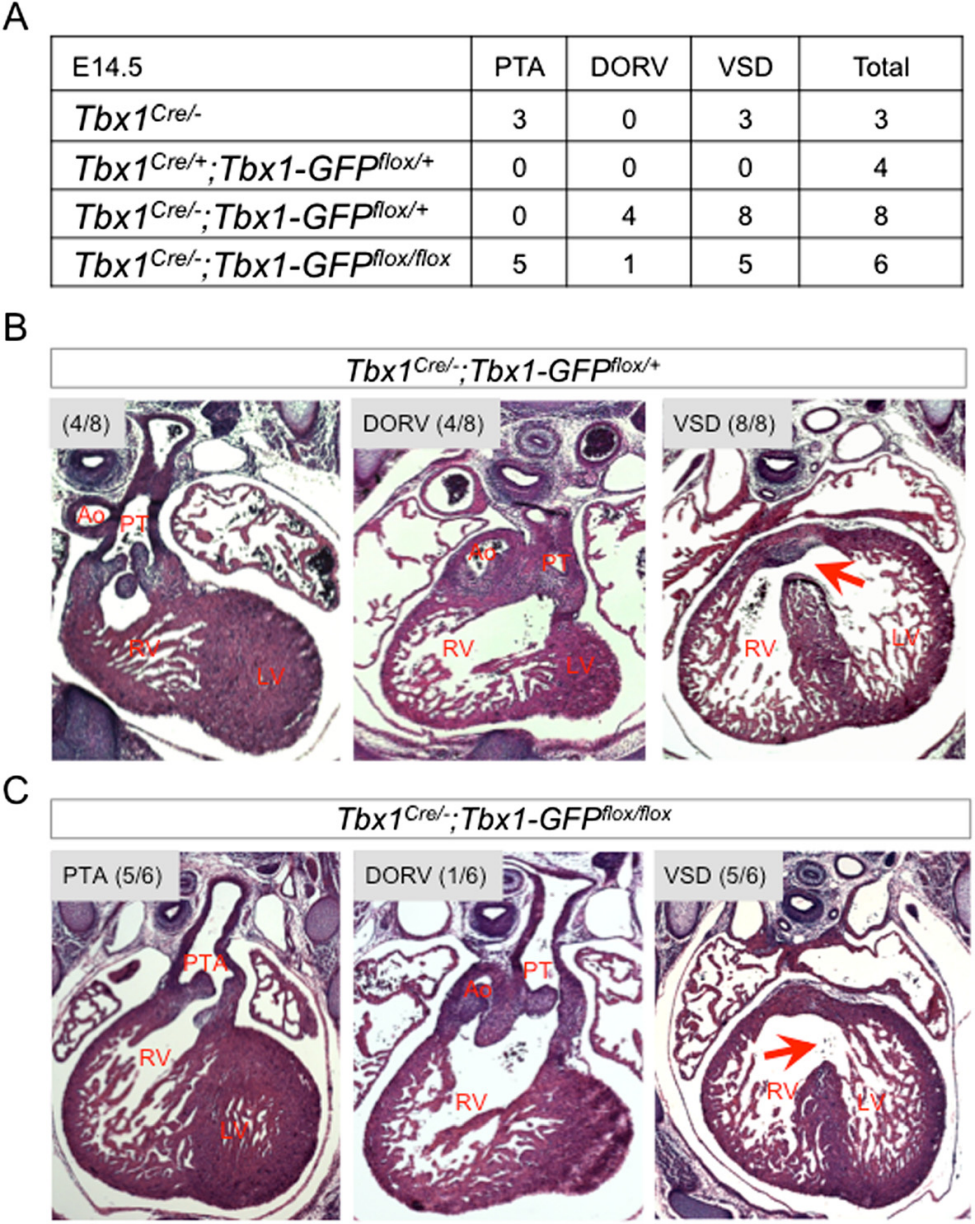
***Tbx1-GFP *can partially rescue endogenous *Tbx1***^***Cre/-***^**loss-of-function.** Histological transverse sections through the heart at E14.5. **(A)***Tbx1*^*Cre/-*^ mice are functionally null and have PTA and VSD. *Tbx1*^*Cre/+*^*;Tbx1-GFP*^*flox/+*^ mutants are viable with normal heart histology. **(B)***Tbx1*^*Cre/-*^*;Tbx1-GFP*^*flox/+*^ mutants exhibit complete rescue of outflow tract septation although half of the embryos have double outlet right ventricle (DORV). **(C)***Tbx1*^*Cre/-*^*;Tbx1-GFP*^*flox/flox*^ mutants all have ventricular septal defect (VSD), primarily with persistant truncus arteriosus (PTA) and one example of DORV.

Next, we wanted to test whether Tbx1-GFP could also rescue the cardiac defects that occur in *Tbx1*^*Cre/-*^ embryos (Figure 6A), all of which have PTA and VSD (n=3) [12]. Analysis of *Tbx1*^*Cre/-*^*;Tbx1GFP*^*flox/+*^ embryos at E14.5 showed a complete rescue of outflow tract septation defects (n=8), although 50% still had a misalignment of the outflow tract resulting in a DORV (Figure 6B). VSD was observed in all *Tbx1*^*Cre/-*^*;Tbx1GFP*^*flox/+*^ embryos. It is not clear if these defects are due to failure to rescue the *Tbx1* null phenotype, or if they are due to overexpression of *Tbx1-GFP* protein (Figure 5D), even though protein expression levels as detected by western blot do not provide information about the functional activity levels of Tbx1-GFP protein. It is also possible that failure of complete rescue is due to the fact that Tbx1-GFP is expressed constitutively under the control of the *Rosa26* promoter and is therefore not subject to temporal regulation. Activation of both *Tbx1-GFP* alleles in *Tbx1*^*Cre/-*^*; Tbx1GFP*^*flox/flox*^ embryos resulted in more severe heart phenotypes including PTA in 5/6 embryos in addition to VSD (Figure 6C) due to further *Tbx1* gene dosage imbalance.

## Conclusions

In summary, we have generated a new mouse model that conditionally expresses a Tbx1-GFP fusion protein. We show that Tbx1-GFP does not act in a dominant-negative manner and can functionally substitute for endogenous Tbx1 during heart and inner ear development by partial rescue of heart and inner ear morphological defects in a *Tbx1*^*Cre/-*^ null background. We cannot completely rule out deleterious effects of the GFP tag protein on the ability of Tbx1 to act as a transcriptional regulator either via direct binding of DNA or via protein: protein interactions as we have not directly tested this. However, ectopic activation of *Tbx1-GFP* by *Foxg1-Cre* and *Pax2-Cre* causes morphological defects of the inner ear that resemble those occurring in BAC316.23 transgenic mice. Taken together with the partial rescue of heart and inner ear defects on a *Tbx1*^*−/−*^ null background, it seems likely that the Tbx1-GFP fusion protein retains functional characteristics of the endogenous Tbx1 protein. As such, the *Tbx1-GFP* mouse line provides a genetic tool that is amenable to probing direct downstream targets of Tbx1 and identifying protein-protein interacting partners of Tbx1 *in vivo.*

## Methods

### Targeting vector

A pTbx1-EGFP-N1 plasmid was generated by cloning *Tbx1* cDNA from a *Tbx1-*TOPO plasmid into pEGFP-N1 (Clontech 6085–1). The following oligonucleotide adaptor sequences were used for an in-frame ligation of the C-terminus of *Tbx1* to the N-terminus of *EGFP*, simultaneously changing the *Tbx1* stop codon from TAG to TTG and introducing NotI and BamHI restriction enzyme sites: 5’-GGCCGCGCCGCCCGGTGCCTACGACTACTGCCCCAGATTG-3’; 5’GATCCAATCTGGGGCAGTAGTCGTAGGCACCGGGCGGCGC-3’. Adaptors were annealed by combining 10 nmol of each oligonucleotide in 200 μL of water and heating to 90°C followed by cooling at room temperature to 40°C. For 20 μL ligation reactions (Roche 1243292, Roche 10799009001), 5 μL of 1:500 diluted annealed adaptors were used. The sequence encoding the *Tbx1-GFP* fusion protein was inserted into the multiple cloning site of pBigT [13]. The loxP-tpA-loxP*-Tbx1-GFP* fragment was excised by restriction enzyme digestion with PacI and AscI then cloned into the pROSA26PA plasmid [13] to generate the final targeting vector (Figure 1). The final targeting vector was linearized with BbvCI and purified by phenol chloroform extraction, then electroporated into WW6 ESC and selection performed with G418. Positively selected ESC clones were plated in duplicate for DNA isolation using the DNeasy Blood and Tissue Kit (Qiagen 69506). DNA was digested overnight at 37°C with EcoRV and Southern blot performed with a previously described 5’ probe [13].

### Western blot

The *Tbx1-GFP* fusion protein was expressed in COS7 cells by transient transfection of the pTbx1-EGFP-N1 plasmid with Polyfect (Qiagen 301105). Protein from whole cell lysate and embryonic tissue was collected in RIPA Lysis Buffer (50 mM Tris HCl pH 7.5, 200 mM NaCl, 1% Triton X-100, 1 mM EDTA, 0.25% deoxycholic acid, protease inhibitor). For tissue samples, the pharyngeal region was isolated from E9.5 embryos and the head, first pharyngeal arch, heart, and caudal part of the embryos (below the fourth pharyngeal pouch) were removed. Tissue was homogenized and lysed in RIPA buffer for 2 hours at 4°C. Samples were run on 7.5 or 10% polyacrylimide gels. Tbx1-GFP fusion protein (≈75 kD) was detected using polyclonal anti-Tbx1 (Zymed, 1:500), anti-GFP (Invitrogen A6455, 1:600), mouse anti-βactin (Abcam ab6276, 1:5,000), donkey anti-Rabbit IgG-HRP (Amersham NA934V, 1:20,000) and sheep anti-Mouse IgG-HRP (Amersham NA931V, 1:5,000) antibodies. Detection was performed using the ECL Western Blotting Detection Kit (Amersham RPN2106) and exposed on Kodak film.

### Mouse models

*Tbx1-GFP* mice were genotyped with the FastStart High Fidelity PCR System (Roche 03553361001) and the following primers: TB3F (5’-CTGCACCACCATCCCTACAA-3’) and GFPR (5’- TGAACTTCAGGGTCAGCTTG-3’) for a 421 bp product from the targeted allele, and RO1F (5’-GCAATACCTTTCTGGGAGTT-3’) and GFP-wtR (5’- CAATGCTCTGTCTAGGGGTT-3’) for a 605 bp product from the wildtype allele. *Foxg1-Cre, Pax2-Cre*, and *Tbx1*^*Cre/+*^ mice were kindly provided by Drs. Jean Hebert, Andrew K. Groves, and Antonio Baldini, respectively.. Embryos were dissected according to date of vaginal plug (E0.5). Embryonic stages <E11.5 were confirmed by counting pairs of somites. Animals were maintained in a 12 hr dark/12 hr light cycle in compliance with the Albert Einstein College of Medicine of Yeshiva University Institutional Animal Care and Use Committee (IACUC). *Tbx1-GFP* mice will become available to the research community upon acceptance of the manuscript.

### RNA *in situ* hybridization

Embryos were fixed in 4% paraformaldehyde (PFA) at 4°C overnight then dehydrated in a methanol series. RNA *in situ* hybridization (ISH) was performed following rehydration to 0.1% PBS/0.1% Tween-20 (PBT) [24]. Anti-sense digoxigenin-labeled RNA probe for *NeuroD*[25] was generated from a plasmid by standard protocol. The RNA probe template for *netrin-1* was generated from amplified E9.5 mouse cDNA using the following primers: Netrin1-Fwd (5’-GGGGAATTAACCCTCACTAAAGGGTGATCCTTGCTCGGATGAGA-3’), Netrin1-Rev (5’-GGGGTAATACGACTCACTATAGGGTTCTTCTCCCGTTGCTGGAA-3’).

Primer sequences introduced T7 RNA polymerase binding sites for generating antisense probes and T3 RNA polymerase binding sites for generating sense probes. For wholemount ISH, we rehydrated embryos to PBT and digested them with Proteinase K. This was followed by washes in Glycine solution and PBT. Embryos were then fixed in 4% PFA/0.2% gluteraldehyde for 15 min on ice followed by PBT washes. Embryos were incubated with RNA probes in hybridization buffer overnight at 70°C then washed in a series of SSC, maleic acid buffer, and PBT washes. Then embryos were incubated overnight at 4°C in antibody buffer with 1:10,000 dilution of anti-Digoxigenin-AP antibody (Roche 11093274910) followed by washes in 0.1% BSA/PBT and AP1 buffer. Staining was performed using BM Purple (Roche 11442074001) followed by fixation in 4% PFA. RNA *in situ* hybridization to *netrin-1* was performed on tissue cryosections according to the David Anderson laboratory protocol. Briefly, tissue sections were fixed in 4% PFA for 20 min at RT and digested with Proteinase K followed by post-fixation in 4% PFA for 15 min. Acetylation was performed in TEA buffer with acetic anhydride followed by washes in PBS and air-drying. Hybridization with RNA probes was done overnight at 68°C in a hyb chamber humidified with 50% formamide/4X SSC. Tissue sections were washed with SSC and PBT then incubated overnight at 4°C in blocking buffer with 1:2,000 dilution of anti-Digoxigenin-AP antibody followed by washes in PBT and AP1 buffer and staining with BM Purple.

### Immunofluorescence and whole mount immunohistochemistry

Embryos were fixed overnight in 4% paraformaldehyde, placed in 30% sucrose/PBS, embedded in O.C.T., then cryosectioned at 12 μm thickness. For immunofluorescence, tissue sections were washed in PBS, permeabilized for 5 min in 0.5% Triton X-100, then incubated in blocking solution (5% serum in PBS/0.1% Triton X-100 i.e. PBT) for 1 hour. Primary antibodies (rabbit anti-mouse pan-laminin 1:200, Chemicon AB2034; rabbit anti-Tbx1 1:500, goat anti-GFP 1:500, Abcam ab6673) were diluted in block and incubated on tissue for 1 hr at room temperature. Sections were washed in PBT then incubated with secondary antibody (Alexa Fluor 568 goat anti-rabbit IgG 1:500; Alexa Fluor 568 donkey anti-goat IgG 1:500) for 30 min. DAPI (1:500) was added to secondary antibody. Sections were washed in PBT mounted in Vectashield hard-set mounting medium (Vector Labs H-1400). For whole mount immunohistochemistry, embryos were fixed overnight in 4:1 methanol/DMSO at 4°C. Endogenous peroxidase activity was blocked in 4:1:1 methanol/DMSO/30% H_2_O_2_ for 6 hrs at room temperature. Embryos were then rehydrated in a methanol series and blocked in 2% milk/0.5% Triton X-100/PBS (PBSMT) for 1 hour. Primary antibody (anti-neurofilament 1:200, DSHB mAb2H3) was diluted in PBSMT and incubated overnight at 4°C. Embryos were washed five times for an hour each in PBSMT at 4°C and then incubated with [HRP]-sheep anti-mouse IgG (Amersham NA931V, 1:500) diluted in PBSMT overnight at 4°C. Embryos were washed again in PBSMT for 5 hrs at 4°C and then 30 min in 0.2% BSA (Sigma A4503)/0.5% Triton X-100/PBS. Staining was achieved with DAB substrate with chromagen (DAKO K3466) for 15 min at room temperature. Embryos were post-fixed in 4% paraformaldehyde overnight at 4°C, dehydrated in a methanol series, then cleared in 1:2 Benzyl alcohol: Benzyl Benzoate (BABB) for imaging and long-term storage.

### Inner ear paintfilling and histology

Embryos were sliced below the forelimbs then fixed in 5% glacial acetic acid, 2% formaldehyde, and 75% ethanol overnight followed by an overnight dehydration in 100% ethanol. Methyl salicylate was then used for clearing of the tissue and long-term storage. Prior to paintfilling, embryos were bisected dorsally and the brain was removed to reveal the inner ear capsule. A micropipette was used to microinject 0.2% correction fluid diluted in methyl salicylate into the inner ear labyrinth. For histological analysis, embryos were fixed in 4% paraformaldehyde overnight at 4°C. They were then dehydrated to 70% ethanol and embedded in paraffin. Tissue sectioning was performed at 10-12 μm thickness. Tissues were cleared in xylene, stained with hematoxylin and eosin (H&E) and then mounted in Permount.

## Competing interests

The authors declare no financial or non-financial competing interests in relation to this manuscript.

## Authors’ contributions

LF performed experiments, analyzed data, and wrote the manuscript. SN generated the Tbx1-GFP fusion plasmid, designed the targeting vector, and commented on the manuscript. MP performed the Southern Blots to screen for targeted embryonic stem cells. AB provided the *Tbx1*^*Cre/+*^ mouse line used for rescue experiments. BEM formulated the project, supervised and funded the experiments, and revised the manuscript. All authors read and approved the manuscript.
